# Genomic variability and immunological aspects involved in response to MPXV infection

**DOI:** 10.3389/fphar.2025.1665830

**Published:** 2025-09-24

**Authors:** Simone La Frazia, Anna Rosa Garbuglia, Silvia Pauciullo, Verdiana Zulian, Paola Del Porto

**Affiliations:** ^1^ Department of Biology, University of Rome Tor Vergata, Rome, Italy; ^2^ Laboratory of Virology, National Institute for Infectious Diseases “Lazzaro Spallanzani” (IRCCS), Rome, Italy; ^3^ Department of Biology and Biotechnology “Charles Darwin”, Sapienza University of Rome, Rome, Italy

**Keywords:** Mpox, genome, pathogen characteristics, transmission, vaccines

## Abstract

Mpox, caused by the monkeypox virus (MPXV), is a zoonotic disease that has gained global relevance after the 2022–2024 outbreak. MPXV exists in two distinct clades: clade I, associated with higher virulence and mortality, and clade II, which demonstrated increased human-to-human transmission and adaptation. Clinically, Mpox presents with rash, fever, and lymphadenopathy, with severe complications in immunocompromised individuals. Genomic surveillance has revealed rapid evolution, partly driven by APOBEC3-mediated mutagenesis, especially within immune-modulating regions. Although smallpox vaccines like MVA-BN and ACAM2000 provide cross-protection against Mpox, the MVA-BN vaccine showed a more favourable safety profile, but variable effectiveness compared to replicating vaccines. Antiviral agents such as tecovirimat and cidofovir have been used off-label, but emerging resistance and limited clinical efficacy highlight an urgent need for MPXV-specific therapeutics. The current epidemiological scenario emphasizes the importance of novel antiviral development and optimized prophylactic strategies to improve clinical outcomes and global preparedness. This review aims to provide a comprehensive overview of the molecular biology, clinical features, the current drugs used to treat Mpox infection and the vaccines used to prevent the infection. It also discussing the limitations of these therapeutic tools and the improvements needed to enhance their efficacy.

## Highlights

• Current antiviral options for monkeypox, including tecovirimat, brincidofovir, and cidofovir, show promising preclinical efficacy but are limited by insufficient clinical data, potential toxicity, and the risk of resistance. Future efforts should prioritize well-designed clinical trials, resistance monitoring, and the development of safer, more effective agents or combination regimens.
• Like other OPXVs, MPXV possess a high capacity for genetic recombination and can incorporate genes from other viruses, making it a candidate for gain-of function (GOF) or research of concern (GOFROC) experiments.
• Second and third generation smallpox vaccines can be used to protect people at high risk of MPXV exposure. The currently available vaccines differ in safety profile and efficacy. Further studies are needed to establish the role of humoral and cellular immune responses in vaccine induced protection.

## 1 Introduction

The monkeypox virus (MPXV) is a double-stranded DNA (dsDNA) virus, belonging to the *Orthopoxvirus* (OPXV) genus within the *Poxviridae* family. This group also includes other notable viruses such as variola virus (VARV, smallpoxvirus), vaccinia virus (VACV), cowpox, camelpox virus, Taterapox virus, and ectromelia virus (World Health Organization, 2024a). The high homology among these viruses (more than 95.5% with smallpox) leads to an immunological cross-reactivity within the genus.

MPXV is the causative agent of Mpox, formerly known as monkeypox, a zoonotic disease characterized by skin lesions, fever, and lymphadenopathy, which can range from mild to severe and may lead to complications, particularly in immunocompromised individuals. The first case of Mpox was identified in 1959 during vaccine-related studies involving a laboratory monkey in Copenhagen, Denmark. However, the first confirmed human case of Mpox was observed in 1970 in a child living in the Basankusu territory of Democratic Republic of Congo (DRC) (Ladnyj et al., 1972).

Genomic analyses have identified two distinct MPXV clades: clade I and clade II.

Prior to the 1980s, the spread of MPXV was limited due to the cross-protection provided by the smallpox vaccination. However, since Mpox is a zoonosis, sporadic cases have been observed in rural areas, where zoonotic transmission from infected animals remains a key route of infection. Natural reservoirs include various African rodents and non-human primates such as apes and monkeys (Li et al., 2025; Sun et al., 2024). From 1970 to 1999, over 500 Mpox cases had been reported in Africa, but most of them (98.7%) occurred in DRC (Brown and Leggat, 2016; Jezek et al., 1987). The first Mpox outbreak outside of the African continent was reported in the United States in 2003. It was caused by the importation of rodents from Ghana (Anderson et al., 2003). Between 2018 and 2021, international travel-related cases were described in countries such as USA, Israel, the United Kingdom, and Singapore with secondary cases among healthcare workers (Vaughan et al., 2018). In May 2022, the greatest outbreak outside endemic area occurred, leading the WHO to declare the event a public health emergency of international concern on July 23, 2022 (Adegboye et al., 2022; Thornhill et al., 2022). As of July 31, 2025, a total of 34386 confirmed Mpox cases and 138 deaths had been reported across 84 countries (World Health Organization, 2025b). The first wave of outbreak was caused by clade II strains, but in September 2024 several cases, which were linked to clade I, were reported in DRC (The Johns Hopkins University, 2024a). This review will explore the general aspects of Mpox infection, including clinical manifestations and its classification as a select agent requiring stringent biosafety indications. We will examine the genome structure, emerging patterns of antiviral resistance to drugs used for the treatment of Mpox, such as Tecovirimat and Cidofovir and vaccination. Moreover, an overview is done on limitations of these therapeutic tools and the improvements needed to enhance their efficacy.

## 2 MPXV genome

The MPXV genome is a linear, double-stranded DNA (dsDNA) of approximately 197 kbp, characterized by covalently closed hairpin termini that lack free 3′ or 5′ ends (Monzón et al., 2024). It encodes over 190 proteins, including polymerases. There are ∼6.4 kb inverted terminal repeats (ITRs) at both extremities of the genome. The genome is divided into three regions: a central core and two flanking arms (left and right), each containing an ITR. The conserved core region genes are essential for viral replication, transcription, and virion assembly (Karagoz et al., 2023).

In contrast, the terminal arms are more variable and encode proteins that modulate host range and pathogenicity (Young et al., 2024). In addition to coding regions, the MPXV genome contains several noncoding regions that play essential roles in viral replication and gene regulation. These include promoter regions, which regulate viral gene transcription by serving as binding sites for viral RNA polymerase and associated transcription factors, and replication origin, which are specific DNA sequences that initiate and control viral genome replication, ensuring accurate and efficient genome duplication during the viral replication cycle (Cheng et al., 2025).

Gene density is high, with most genes arranged compactly and intergenic regions rarely exceeding 100 bp (Karagoz et al., 2023). MPXV, like other members of the *Poxviridae* family, exhibits a relatively low mutation rate, estimated at approximately 10^-5^ to 10^-6^ substitutions per nucleotide per replication cycle. However, this rate is more than three orders of magnitude (i.e., over 1000-fold) higher than that of host cellular DNA polymerases (Karagoz et al., 2023; Mohanto et al., 2023). The Central coding region is a highly conserved segment comprising approximately 138 kbp (Chen et al., 2005), that encodes essential proteins for viral replication, transcription, virion assembly, and host cell interactions (Yu et al., 2024).

This region included core genes encoding viral RNA and DNA polymerases (such as F8), characteristic of a DNA virus with cytoplasmic transcription and replication. Viral DNA polymerase is a complex comprising processivity factor A22, DNA glycosylase E4, and phosphoprotein H5 (Wang et al., 2023; Xie et al., 2025). Other proteins are involved in DNA replication, such as helicase-primase (E5), single-strand binding proteins (I3), and topoisomerases, which facilitate the unwinding and stabilization of the viral genome during replication (Wang et al., 2023). Moreover, core genes encode several virion core proteins, including the mRNA capping enzyme and structural proteins that contribute to the formation of the viral core and facilitate the assembly of new virions.

This region of the MPXV genome also encodes proteins essential for viral entry and virion assembly: A27L, B5R, and F13L are membrane-associated proteins that play critical roles in viral entry, fusion, and budding (Hughes et al., 2014; Sagdat et al., 2024). The B5R glycoprotein is the MPXV VAP (Viral Attachment Protein) involved in receptor binding, which allows the virus to enter host cells by interacting with glycosaminoglycans (GAGs) on the target cell surface (Sagdat et al., 2024; Tang et al., 2023). Adjacent to central coding region, the MPXV genome has the ends of variable regions containing inverted terminal repeats (ITRs). These regions contain genes involved in immune modulation, pathogenicity, and host range determination, collectively influencing virulence and rapidly enabling the virus to adapt to new hosts (Karagoz et al., 2023).

At least four open reading frames (ORFs) have been identified in ITR (Kugelman et al., 2014).

In particular, the ITRs represent hotspots of genetic variability within the variable regions of OPXV genomes and are essential for the replication and packaging of the viral genome, promoting circularization that facilitates efficient cytoplasmic replication and the formation of new virions (Ji et al., 2024).

**FIGURE 1:**
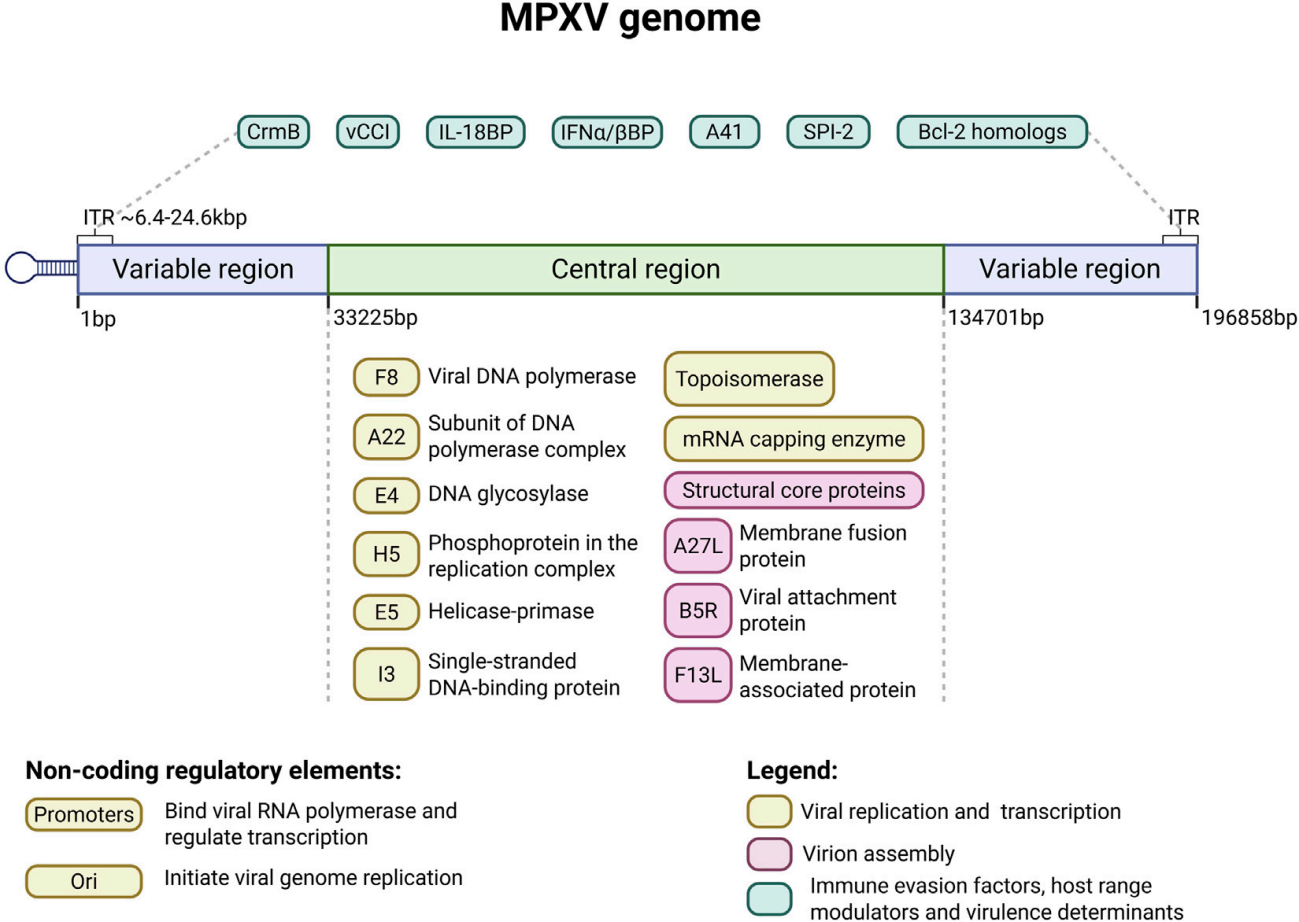
Schematic representation of the monkeypox virus (MPXV) genome. The MPXV genome is a linear, double-stranded DNA molecule of approximately 197 kilobase pairs (kbp), flanked by inverted terminal repeats (ITRs) at both ends. It is divided into a highly conserved central core region (∼138 kbp; shown in light green) and two variable terminal arms (shown in blue). The central region encodes essential genes for viral replication, transcription, and virion assembly, including DNA and RNA polymerases (e.g., F8), helicase-primase (E5), single-stranded DNA-binding protein (I3), topoisomerases, and membrane-associated proteins (A27L, B5R, F13L) involved in viral entry and morphogenesis. The terminal arms, including the ITRs (∼6.4–24.6 kbp), contain genes involved in host range determination, immune evasion (e.g., CrmB, vCCI, IL-18BP, IFNα/βBP, A41), and anti-apoptotic functions (e.g., SPI-2 and Bcl-2 homologs: A47R, B13R, C6L, D11L, and P1L). Notably, the ITRs contain genes that can be deleted or duplicated through genomic rearrangements—such as those observed during the 2022 outbreak—that affect both the left and right terminal arms, contributing to viral adaptation and plasticity (Otieno et al., 2025). Created on Biorender.

MPXV employs sophisticated strategies to evade the host’s immune system, contributing to its capacity for persistent infection. One of the mechanisms of immune response evasion involves the production of cytokine-binding proteins that sequester and neutralize host cytokines, thereby dampening immune activation, including cytokine response modifier B (CrmB), viral chemokine inhibitor (vCCI) and A41 (Jiang et al., 2024; Jones et al., 2008). Furthermore, MPXV encodes IL-18 binding protein (IL-18BP) and effectively inhibits IL-18-induced pro-inflammatory NF-κB activation, reducing IFN-γ production and altering NK cell and T cell activation (Yi et al., 2024). Unlike vaccinia virus (VAXV), MPXV produces truncated, inactive orthologs of the interferon antagonist proteins E3L and K3L that interfere with host interferon signaling pathways; hence, antiviral defences and viral persistence are promoted mainly by expression of the soluble type I interferon binding protein (IFNα/βBP) (Guan et al., 2023) (Figure 1).

Additionally, MPXV expresses anti-apoptotic factors, including the Bcl-2 homologs A47R, B13R, C6L, D11L, and P1L, and the caspase-1 and caspase-8 inhibitor protein SPI-2, which suppress programmed cell death in infected cells, prolonging their viability and promoting viral replication (Alakunle et al., 2024; Lu et al., 2023; Lum et al., 2022). MPXV encodes proteins that enable binding to host cell receptors like CD8 and CD4 on T-cells, influencing the virus’s host range (Hammarlund et al., 2008). Genes involved in immune evasion contribute to strain-specific virulence.

Comparative analyses across the OPXV genus reveal that ITRs are present in most species (over 90%), with only minor sequence differences observed among homologous segments. Specifically, analyses of 825 MPXV genomes identified ITRs in 823 samples, highlighting their conserved and indispensable role in the virus’s life cycle (Yu et al., 2024). The ITRs have significant genomic plasticity, characterized by dynamic events such as gene duplications, deletions, and the proliferation of tandem repeat sequences. Brinkmann and colleagues demonstrated that significant structural variations were observed during the 2022 Mpox outbreak, including extensive duplications and deletions of genetic material. These genomic rearrangements contributed to an expansion of the ITR regions from approximately 6.4 kbp to nearly 24.6 kbp. Specifically, duplications spanned up to 18.2 kilobases, while deletions reached lengths of up to 16.9 kilobases. Such modifications predominantly impacted genes implicated in immune evasion and host range adaptation, underscoring the evolutionary plasticity of the viral genome (Brinkmann et al., 2023). Unique 16-nucleotide tandem repeats have been identified within the ITRs of the MPXV genome. These repeat elements exhibit clade-specific variation in copy number and appear exclusive to MPXV, with no homologous sequences detected in other Poxviruses or human or rodent genomes (Desingu et al., 2023). This clade-dependent polymorphism suggests a possible role for these repeats in MPXV genomic plasticity and evolution, with potential implications for understanding viral diversity and informing targeted vaccine design (Desingu et al., 2023).

## 3 Strain variability

Since the first diagnosed human case in the Democratic Republic of Congo in 1970, MPXV has become endemic in Central and Western Africa (Kantele et al., 2016). Genetic characterization divides MPXV into two major clades with distinct geographic distributions and pathogenic profiles: clade I (previously known as the Congo Basin or Central African clade) and clade II (formerly the West African clade) (Otieno et al., 2025; Yu et al., 2024).

Clade I viruses, including the ZAI-96 strain, are linked to increased virulence and mortality rates that can reach 10% (1.4%–10% range), potentially due to unique genetic elements involved in apoptotic pathways that enhance the pathogenicity (Alakunle et al., 2024; Lu et al., 2023; The Johns Hopkins University, 2024b). Conversely, clade II strains, such as SL-V70, COP-58, and WRAIR-6, cause less severe disease and exhibit mortality rates up to 3.6% (0.1%–3.6% range) (Monzón et al., 2024; The Johns Hopkins University, 2024b; Zhang et al., 2024). Clade II is further subdivided into subclades IIa and IIb, the latter was responsible for the outbreak from 2022 to 2024, originated in Nigeria (Cevik et al., 2024; The Johns Hopkins University, 2024b). Both clade I and subclade IIa primarily circulate endemically within animal reservoirs, which remain incompletely characterized but likely include rodents and nonhuman primates.

Notably, the 2022 Mpox outbreak revealed a significant shift in epidemiology with the emergence of a novel grouping designated clade IIb that exhibit ongoing adaptive evolution (Isidro et al., 2022). This clade, particularly the B.1 lineage, has become the dominant circulating strain globally, demonstrating enhanced human-to-human transmissibility outside endemic regions (Yu et al., 2024). Genomic comparisons demonstrate that MPXV clades differ primarily in genes involved in host interaction, including viral entry, immune evasion, and antigenic diversity (Molteni et al., 2023). The 2022 outbreak strains are distinguished from ancestral MPXV isolates by approximately 46 single-nucleotide polymorphisms (SNPs), underscoring substantial molecular evolution (Yu et al., 2024). Notably, the B.1 lineage displays an elevated substitution rate estimated to be six to twelve-fold greater than the mutation rate observed in isolates from 2018 to 2019 suggesting accelerated viral evolution (Lu et al., 2023).

Comparative analysis of the genomic sequences of MPXV strains from the Congo Basin and West Africa revealed approximately 99% nucleotide identity within each geographic cluster, but only about 95% identity when comparing strains across these regions (Rabaan et al., 2023).

The global outbreak of Mpox in 2022 prompted a rapid and extensive virological response, including the large-scale sequencing of viral genomes from numerous cases across different regions (Brinkmann et al., 2024; Isidro et al., 2022). These genomic investigations uncovered a range of mutations, particularly within genomic regions associated with viral replication and immune evasion. Mutational changes in these regions have been hypothesized to influence several virological and epidemiological parameters, including host range, vaccine responsiveness, and virulence (Young et al., 2024). Specifically, alterations in the genome may affect zoonotic transmission dynamics, enabling the virus to infect a broader range of species or enhance human-to-human transmissibility (Pan et al., 2023).

The ITRs are especially susceptible to editing by host-derived cytidine deaminases, such as members of the APOBEC3 (apolipoprotein B mRNA-editing enzyme, catalytic polypeptide-like 3) family (Brinkmann et al., 2023; O’Toole et al., 2023). APOBEC3 enzymes preferentially introduce G to A and C to T mutations within single-stranded DNA, particularly targeting cruciform DNA structures that can form in palindromic or repetitive regions such as ITRs (Van Norden et al., 2024). In the 2022 MPXV outbreak, a substantial percentage of observed SNPs were located within these ITR domains, suggesting that APOBEC3-mediated editing represents a significant factor in the evolution of MPXV during human infection (Fouad and Elsaid, 2023). Concerning this, recent studies have identified mutations in the APOBEC-3 gene, suggesting an adaptation of the virus to humans (Fouad and Elsaid, 2023). Further support for APOBEC3 activity in MPXV evolution comes from mutational signatures identified in recent genomic studies. The elevated frequency of TC>TT and the corresponding GA>AA substitutions has been particularly pronounced in MPXV clade IIb, which has demonstrated an increased substitution rate relative to other OPXV (Alakunle et al., 2024; Otieno et al., 2025; Yu et al., 2023). Despite the adaptive pressures exerted by the human immune system, MPXV continues to exhibit considerable genetic diversity, reflecting complex transmission dynamics and long-term maintenance in animal reservoirs. This is particularly evident when comparing viral genomes from different geographical clades.

Otieno and colleagues found that clade I is believed to be divergent from a common ancestor around 1917, and was detected in regions such as Sudan, indicating sustained circulation in animal reservoirs (Otieno et al., 2025). Notably, genomic analysis of the Sudan isolates revealed approximately 10.5 kbp of duplication and a TC>TT transition mutation rate, consistent with that observed in animal hosts and much lower than that expected from sustained human transmission, suggesting that this lineage has predominantly evolved within a nonhuman host for several decades, reinforcing the importance of zoonotic reservoirs in the persistence and re-emergence of Mpox (Otieno et al., 2025).

The geographic and temporal distribution of MPXV clades further illustrates the complex epidemiology of the virus, and the persistence of genetically distinct clades in zoonotic reservoirs highlights the persistent risk of spillover events (Otieno et al., 2025).

## 4 Clinical manifestation

Mpox, unlike smallpox, is a zoonotic disease that can be transmitted to humans through different animals. These include rodents (tree squirrels, dormice, African squirrel, Gambian Kangaroos), rabbits, prairie dogs (Bunge et al., 2022; Hutson et al., 2009), apes, and monkeys, even though the natural reservoir remains unknown. Studies in animal models, such as mice and monkeys, have demonstrated the clade I strains are more virulent than those of clade II, consistent with observed clinical outcomes in humans (Falendysz et al., 2023). The clinical presentation of both clades is broadly similar, with severity being the main distinguishing factor (Kipkorir et al., 2022). The animal-to-human transmission generally results from direct contact with skin lesions of infected animals or through the consumption of undercooked meat of infected animals (Angelo et al., 2019; Grant et al., 2020). Human-to-human transmission occurs through direct contact with virus particles in lesion secretion and body fluids (Grant et al., 2020). MPXV can also be transmitted via respiratory droplets during prolonged close contact, and through fomites such as contaminated clothing, bedding, and medical instruments (e.g., needles). Given that the virus is present in oropharyngeal secretions from the onset of clinical symptoms, extended exposure to respiratory droplets represents an additional route of transmission. Exudative lesions, blood, and other body fluids serve as sources of infection in both zoonotic and interhuman transmission. Secondary human-to-human transmission has been well-documented, such as during the 1996–1997 Central African outbreak, where 73% (65/89) of patients reported close contact with a confirmed case 7–21 days before symptoms onset (Centers for Disease Control and Prevention, 1997). Nosocomial transmissions were also documented in African outbreak (Nakoune et al., 2017) and in the United Kingdom, where healthcare workers were exposed while handling contaminated bedding without appropriate personal protective equipment (PPE) (Vaughan et al., 2020). In Human primates, infection can be initiated by intrabronchial administration of 5 × 10^4^ plaque forming units (PFU) (Johnson et al., 2011). MPXV can be isolated from various types of clinical samples, each typically containing at least 10^6^ viral copies (Rheinbaben et al., 2007).

The infectious dose of MPXV differs between clade I and clade II and depends on the route of exposure and the animal model used. Experimental studies have demonstrated that clade I strains are generally more virulent than clade II strains. For example, in prairie dogs infected intranasally, clade I viruses showed greater pathogenicity compared to clade II viruses with the same viral doses administrated (Hutson et al., 2009).

In a separate study, the infectivity and pathogenesis of clade I, clade IIa, and clade IIb MPXV strains were compared using the CAST/EiJ mouse model. Animals were infected via intranasal and intraperitoneal routes with viral doses ranging from 10^2^ to 10^5^ plaque-forming units (PFU), depending on the administration route. Results demonstrated that clade I virus exhibited significantly higher virulence compared to clade IIa and IIb strains, as evidenced by increased morbidity, mortality, and viral dissemination within infected tissues. These findings highlight the differential pathogenic potential among monkeypox virus clades (Americo et al., 2023).

Although these findings underscore clade-dependent differences in virulence, it is important to note that the minimum infectious dose (ID_50_) in humans remains undetermined. No studies to date have established a definitive human infectious dose for either clade I or clade II.

In humans the incubation period ranges from 5 days to 21 days, with a median of 6–13 days (Guzzetta et al., 2022; Miura et al., 2022). However, infected individuals may be contagious 1–4 days before the onset of symptoms (Sun et al., 2024). The symptomatology is consistent between clade I and clade II, with severity being the primary differentiator (Kipkorir et al., 2022). Initial symptoms include muscle aches, headaches, fatigue, and fever. The characteristic rash and mucosal involvement usually emerge within 1–3 days following the onset of fever, typically affecting the face, back, soles of the feet, and palms (Falendysz et al., 2017). During the 2022 outbreak in non-endemic geographical areas, most of Mpox cases occurred among men have sex with men (MSM), where the sexual contacts represented the main route of transmission. In these patients, atypical manifestations such as genital ulcers and perianal lesions were frequently observed, along with a higher prevalence of inguinal lymphadenopathy compared to axillary or cervical lymph node involvement (Antinori et al., 2022; Bragazzi et al., 2023; Ferré et al., 2022; Mitjà et al., 2023b; Ogoina, 2022).

The virus penetrates the body through skin lesion or mucous membranes and replicate in keratinocytes, fibroblasts, and endothelial cells (Mitjà et al., 2023b). From the initial infection site, MPXV can spread to draining lymph nodes, where the virus can replicate and subsequently reach spleen and liver. Some patients also present oropharyngeal involvement, including oral ulcers and tonsillitis, and ocular symptoms such as conjunctivitis and blepharitis (Damon, 2011). Serious complications may include bronchopneumonia and sepsis (Petersen et al., 2019). Although rare, cardiovascular complications, such as myocarditis, viral pericarditis, heart failure, and arrhythmias, have been reported. MPXV can access the central nervous system (CNS) via the olfactory epithelium or infected circulating monocytes/macrophages, potentially resulting in encephalitis (Sepehrinezhad et al., 2023). Additional complications may include conjunctivitis, diarrhea, and vomiting (McCollum and Damon, 2014). Moreover, new complications in 2022 outbreak had been described like perineal lesions and lymphadenopathy (Damon, 2011; Liu et al., 2023), paraphimosis, whitlow, and proctitis (Cherfan et al., 2023; Grau Echevarría et al., 2023; Pinnetti et al., 2024).

In most cases, Mpox is a self-limiting illness with a favourable prognosis, resolving within 2–4 weeks. However, the disease may take a more severe course in young children, pregnant individuals, and those with compromised immune systems (Liu et al., 2022). Moreover, some different clinical manifestations had been observed between different MPXV clades and lineage. For example, clade Ia, IIa and IIb lineage A show a generalized lymphadenopathy (Pittman et al., 2023), whereas in clade IIb lineage B.1 cases more frequently lymphadenopathy is localized in specific areas such as genital, anal, and oral region (Mitjà et al., 2023b; Thornhill et al., 2022). Clade IIb generally does not lead to severe complications except in immune compromised patients (Mitjà et al., 2023a). The higher severity of clade I infection could be correlated with high capacity of clade I to induce marked dysregulation of host cytokines, causing a strong inflammatory response and tissue damage (Huang et al., 2024). Furthermore, clade I replicates more efficiently in human cells. This allows to virus to replicates faster in comparison to clade II and to have a wide dissemination in short time (Americo et al., 2023).

## 5 MPXV as select agent

MPXV is classified in risk group 3 (Government of Canada, 2023). The recommendations for packing, shipping, inactivation methods, and managing of waste contaminated with MPXV differ depending on the viral clade. The clade I is classified as a Select Agent (SA), whereas specimens infected with clade II are not subject to SA regulations (Centers for Disease Control and Prevention, 2025a). Specimens containing MPXV clade II can be packed and shipped as Category B infectious substances (UN3373, Biological substances, Category B) and do not require a DOT (DOT Transporting Infectious substance safety) special permit under the DOT guidelines for transporting infectious substances (Centers for Disease Control and Prevention, 2024a). Routine diagnostic activities on specimens suspected to contain MPXV should be conducted in Biosafety Level 2 (BSL2) Laboratory. The recommended precautions that should be included are: eye protection, NIOSH-approved respirator with N95 filters or higher, double gloves, limiting the number of laboratory personnel involved in specimen manipulation, and working in laboratory with directional air flow (Meechan and Potts, 2020a; 2020b).

The wastewater samples suspected to contain MPXV should be pasteurized (60 °C for 1 h) before processing. However, all culture-based testing for monkeypox virus must be carried out in BSL3 Laboratory (i.e., seroneutralization assays) (Centers for Disease Control and Prevention, 2024b).

Although the case fatality rate of Mpox (up to 10% for clade I) is lower than that of smallpox (up to 30%), several factors could contribute to MPXV emerging as a serious viral pathogen representing a potential public health risk:

• Eradication of Mpox is challenging due to its zoonotic nature;
• A large portion of the global population under 40 years of age has not been vaccinated against smallpox and is therefore susceptible to MPXV infection;
• Currently circulating MPXV strains had been adapted to human (Zhang et al., 2023);
• The ongoing Mpox outbreak has facilitated the global diffusion of MPXV strains, that could potentially be manipulated to engineer more virulent strains using genetic biotechniques.

OPXVs are known to have a great capacity to accommodate foreign DNA. As early as 1993, a team of Russian researchers created a recombinant vaccinia virus containing an inserted DNA copy of 26S RNA from Venezuelan equine encephalomyelitis (VEE) virus. The VEE RNA was inserted into the thymidine kinase gene of the vaccinia virus (Sviatchenko et al., 1993). In 1980, a recombinant poxvirus was constructed by inserting hemagglutinin gene from influenza virus (Panicali et al., 1983). One of the main objectives of engineering poxviruses has been the development of new vaccines. For instance, a copy of the VP24 gene from Ebola virus or a gene encoding the envelope (E) protein of Japanese encephalitis virus was inserted into the vaccinia virus genome (Cheshenko et al., 1997; Shchelkunov et al., 1993). The resulting recombinant viruses were also very stable. These experiments also revealed that inactivation of the E7R gene did not affect the known biological properties of the virus but could contribute to the development of attenuated viral strains. Furthermore, murine interleukin-4 (IL-4) was inserted into the genome of ectromelia virus (mousepox virus) and expressed during infection. Expression of IL-4 by a thymidine kinase-positive strain of ectromelia virus suppressed both natural killer (NK) cell and cytotoxic T lymphocyte (CTL) cytolytic responses, as well as the expression of the interferon-gamma. The mice infected with this IL-4 expressing virus developed symptoms of acute mousepox accompanied by high mortality (Jackson et al., 2001). Even though MPXV has not met the definition of Potential Pandemic Pathogen (PPP), because it does not spread easily, like other OPXVs, MPXV possesses a high capacity for genetic recombination and can incorporate genes from other viruses, making it a candidate for gain-of-function (GoF) or research of concern (GOFROC) experiments. In 2015, Dr. Moss, one of the leading experts in the study of OPXVs, conducted experiments of transferring different genes from a clade II strain of MPXV into the genome of a more virulent clade I strain to assess whether the genes of clade II could reduce virulence (Kaiser, 2022; Kupferschmidt, 2022; Moffit and Howell, 2024). The resulting recombinant virus retained the same level of virulence, indicating that the clade II genes did not attenuate the pathogenicity of clade I. In the second step of experiments, Moss proposed to transfer clade I genes into the genome of a clade II strain to evaluate whether increased virulence could be conferred to the more transmissible clade II strain. Although this experiment was approved by the NIH, it is not clear whether it was ever performed (The United States House of Representatives, 2024). If conducted, this research could result in the creation of a clade II strain with both higher transmissibility (R0>1) and enhanced virulence, characteristics which would elevate it to the status of PPP, representing a great danger for the community. For these reasons, the Federal Government banned these experiments in May 2024.

These findings highlight the need for implementing infection programme prevention by enforcing biosecurity measures to prevent both new variants spreading and to speed the detection of genetically modified MPXV strains. The genomic sequencing is a key tool in the detection of exiting or emerging variants and should be improved with rapid and reliable instrument such as MinION, that is already used in genome surveillance where the resources are limited.

## 6 Antiviral therapy: current limitations, pharmacoresistant mutants, and new drugs

Currently, there are no FDA-approved therapies for Mpox specifically. However, several FDA-approved smallpox antivirals such as cidofovir, brincidofovir, and tecovirimat have been used in Mpox cases (as off label use). Although their efficacy against MPXV in humans has yet to be confirmed, these antivirals are used for immunocompromised individuals, children, pregnant or lactating women, and patients with lesions in sensitive areas such as the mouth, eyes, or genitals (Karagoz et al., 2023).

Cidofovir (CDV) is a non-cyclic nucleoside analogue of deoxycytidine monophosphate with broad-spectrum activity against DNA viruses, including MPXV. It is phosphorylated by host kinases to its active diphosphate form (CDV-PP), which inhibits viral DNA polymerase by competing with dCTP, leading to incorporation into viral DNA and chain termination (Andrei et al., 2006). Unlike other antivirals like acyclovir, CDV does not require viral thymidine kinase, maintaining efficacy against resistant strains (Paintsil and Cheng, 2009). *In vitro* and preclinical studies confirm its activity against MPXV, although clinical use is limited. Due to its nephrotoxicity, it must be co-administered with probenecid and adequate hydration (De Clercq, 2002). In patients with renal insufficiency, it may lead to nephrotoxicity (Lu et al., 2023). *In vivo* studies (rats, rabbits, primates) showed CDV is mainly limited by dose-dependent nephrotoxicity, which affects the proximal tubules. Oral coadministration of probenecid protects against CDV-induced nephrotoxicity (Lacy et al., 1998). It has also shown ocular toxicity (hypotonia, retinal damage) in guinea pigs or rabbits and carcinogenicity (mammary gland) in rats (European Medicines Agency, 2025; Taskintuna et al., 1997). Reproductive toxicity includes testicular atrophy and embryotoxicity in rodents.

During the outbreak of MPXV in 2022, it was rapidly repurposed for off-label use, particularly in severely immunocompromised individuals (Lu et al., 2023; Siegrist and Sassine, 2023). As with human papillomavirus lesions, CDV has also been used topically in MPXV lesions; as well as for cytomegalovirus infections in AIDS patients, CDV has also been used systemically for MPXV (Mertens et al., 2016; Titanji et al., 2024; Yin et al., 2022). In patients with renal insufficiency, it may lead to nephrotoxicity (Lu et al., 2023). CDV remains a therapeutic option in severe or refractory MPXV infections when other antivirals are unavailable or unsuitable. Furthermore, CDV resistance in vaccinia virus was associated with A314T and A684V mutations on the viral DNA polymerase at the exonuclease and palm subdomain levels, respectively (Andrei et al., 2006). The alanine residues at positions 314 and 684 are also conserved in the amino acid sequence of the DNA polymerase of MPXV, suggesting that resistance to this antiviral in MPXV could potentially emerge through analogous mutational mechanisms (Kannan et al., 2022).

Brincidofovir (BDV), also known as CMX001 or Tembex, is an oral lipid prodrug of cidofovir, specifically developed for higher bioavailability and lower nephrotoxicity. After absorption, the lipid portion facilitates cellular uptake and is cleaved intracellularly to release CDV, which is then phosphorylated to its active diphosphate form. Like CDV, BDV inhibits viral DNA polymerase by acting as a competitive analogue of deoxycytidine triphosphate (dCTP), leading to chain termination during viral DNA replication. Brincidofovir therapy offers partial protection and reduces viral load in lethal challenge with MPXV clade II a (Prévost et al., 2024). Compared to CDV, BDV has reduced renal toxicity but has been associated with gastrointestinal side effects, particularly diarrhoea and hepatotoxicity with increased liver enzymes, which may limit tolerability in some patients. Despite limited clinical data in MPXV, CDV remains a potential oral treatment option, particularly when intravenous administration of CDV or tecovirimat is not feasible (Centers for Disease Control and Prevention, 2025b; Lu et al., 2023). Carcinogenicity (e.g., squamous cell carcinoma) was observed in animal studies at sub-therapeutic exposures (Elsevier Health, 2025).

Among the guanosine analogues, ribavirin, a known inhibitor of viral replication of both DNA and RNA viruses, and KAY-2-41 were tested *in vitro* on MPXV or other OPXV; both were effective against resistant CDV mutants (Duraffour et al., 2014; Graci and Cameron, 2006; Lu et al., 2023).

Kannan and colleagues identified ten mutations in the replication complex (RC) present in almost all 2022 genomes, including two in F8L (catalytic subunit of the RC) and two in G9R (processivity factor) (Kannan et al., 2022). Dose-limiting toxicity is related to hemolytic anemia, occurring in 10%–30% of patients, especially during prolonged treatment or in combination with interferon (Russmann et al., 2006).

Tecovirimat (ST-246, TPOXX) is an antiviral agent approved by the FDA for the treatment of smallpox and authorized for MPXV treatment (Russo et al., 2021; U.S. Food and Drug Administration, 2024). It targets the highly conserved VP37 protein, encoded by the F13L gene, which is essential for the envelopment of intracellular mature virions by trans-Golgi-derived membranes to form enveloped virions released from host cell (Blasco and Moss, 1991; Karagoz et al., 2023; Lu et al., 2023). By inhibiting VP37, tecovirimat disrupts the maturation and release of intracellular mature virions without affecting viral replication, thereby limiting intercellular spread (Lu et al., 2023). Preclinical and clinical data have demonstrated potent *in vitro* and *in vivo* activity against MPXV, with observational studies and case reports, particularly in immunocompromised individuals, indicating favourable outcomes and good tolerability (Centers for Disease Control and Prevention, 2025b; Frenois-Veyrat et al., 2022; Postal et al., 2025; Warner et al., 2022). In phase III clinical trials in healthy volunteers, no serious adverse events related to treatment were reported. Tecovirimat is a weak enzyme modulator (CYP3A4, CYP2C8, CYP2C19), with a low risk of clinically relevant interactions (Li et al., 2025). Tecovirimat is metabolized via amide hydrolysis and glucuronidation by uridine diphosphate glucuronosyltransferase 1A1 (UGT1A1) and uridine diphosphate glucuronosyltransferase 1A4 (UGT1A4), reaching steady state in 4–6 days. Its half-life is about 21 h after 600 mg oral dosing and 19 h after 200 mg intravenous (IV) dosing (Mishra et al., 2025).

However, recent randomized controlled trial results in the DRC showed no significant reduction in lesion duration in clade I MPXV infections (PALM007 Writing Group et al., 2025; Van Dijck et al., 2024). Resistance, primarily due to mutations in the F13L gene, has emerged in patients receiving prolonged treatment, and cases of transmitted resistance have been documented (Smith et al., 2023). In fact, reported single nucleotide variations such as H238Q, P243S, N267D, A288P, A290V, A295E, and I372N alter key hydrophobic pockets or binding regions of the VP37 protein, thereby reducing the ability of tecovirimat to inhibit viral replication (Chenchula et al., 2025). However, the results regarding the efficacy of tecovirimat are conflicting, as reported in several clinical trials (Cohen, 2025; Postal et al., 2025).

In addition, NIOCH-14, a prodrug of tecovirimat, showed antiviral effects against OPXV in *in vitro* and *in vivo* studies (Lu et al., 2023; Mazurkov et al., 2016) (Table 1).

**TABLE 1:** Summary of antiviral drugs used or repurposed for the treatment of Mpox.

Drug	Target/strategies	Approved indication	Use for Mpox	Administration in humans	References
Cidofovir	Nucleoside analogue; inhibits viral DNA polymerase	Approved for the CMV retinitis in AIDS patients	Off-label systemic/topical for Mpox	Intravenous; topical	Siegrist and Sassine (2023), Titanji et al. (2024)
Brincidofovir	Oral lipid prodrug of cidofovir; inhibits viral DNA polymerase	FDA-approved for smallpox	Off-label for Mpox	Oral	Centers for Disease Control and Prevention (2025b), Siegrist and Sassine (2023)
Tecovirimat	Inhibits VP37; blocks virion maturation and release	FDA-approved for smallpox	Off-label use for Mpox	Oral or intravenous	Centers for Disease Control and Prevention (2025b), PALM007 Writing Group et al. (2025), Postal et al. (2025), Russo et al. (2021)
NIOCH-14	Prodrug of tecovirimat	Not FDA-approved; licensed in the Russian Federation for treatment of smallpox, Mpox, and cowpox	Preclinical phase	Not applicable	Mazurkov et al. (2016)
Ribavirin	Guanosine analogue; inhibits replication of RNA and DNA viruses	FDA-approved for HCV and RSV	*In vitro* only^a^	Oral or intravenous^b^	Graci and Cameron (2006)
KAY-2-41	Guanosine analogue; inhibits viral replication	Not FDA-approved	*In vitro* only^a^	Not applicable	Duraffour et al. (2014), Lu et al. (2023)
PA104 and Imatinib	Block viral egress	PA104 not approved as standalone treatment; Imatinib approved for several types of cancer	*In vitro* only^a^	Oral^b^	Lu et al. (2023), Priyamvada et al. (2020), Reeves et al. (2011), Reeves et al. (2005)

^a^ This drug is tested against MPXV only *in vitro*.

^b^ This administration is referred to therapy against other viruses or diseases.

The table lists the main pharmacological agents with demonstrated or potential efficacy against Mpox diseases, including their molecular targets or mechanisms of action, routes of administration, approved clinical indications, type of use in Mpox disease (e.g., authorized, off-label, *in vitro*), and relevant literature references. Only compounds mentioned in the main text are included.

During MPXV replication, crescent-shaped membranes form and mature into immature virions (IVs) within viral factories (Barreto-Vieira et al., 2025; Hong et al., 2024). Several antiviral agents disrupt virus assembly, maturation, and release (Lu et al., 2023). PA104 and drugs like imatinib mesylate (STI-571, Gleevec) inhibit actin tail formation, blocking viral egress and showing anti-OPXVs activity *in vitro* (Lu et al., 2023; Priyamvada et al., 2020; Reeves et al., 2005, 2011). EGFR inhibitor gefitinib (Iressa) and MEK inhibitors also impaired OPXV spread (Brayman et al., 1990; Lu et al., 2023; Sliva and Schnierle, 2007).

The viral protein A36R is essential for intercellular transmission and EEV release (Lu et al., 2023). Miah and colleagues identified by virtual screening three peptides that bind A36R with high affinity and potentially effective against MPXV (Lu et al., 2023; Miah et al., 2022).

Overall, current antivirals such as tecovirimat and brincidofovir show efficacy both *in vitro* and *in vivo*, but robust clinical data in MPXV patients are limited. Tecovirimat is usually well tolerated with mild side effects, while brincidofovir is limited by gastrointestinal and hepatic toxicity. The nephrotoxicity of cidofovir limits its routine use. Resistance mutations have been documented *in vitro*, especially to tecovirimat, which targets the viral F13L gene, making resistance monitoring necessary.

Future research should focus on large clinical trials, combination therapies to reduce resistance and toxicity, new antivirals with improved safety, pharmacokinetic studies, and genomic surveillance of resistance.

Combination therapies may offer synergistic effects, enabling comparable efficacy at lower doses while reducing the frequency and severity of adverse events. Furthermore, they represent a promising strategy to prevent the emergence of drug resistance, which has already become a critical concern for tecovirimat in Mpox patients and may also affect cidofovir and brincidofovir (Smee et al., 2002). Because some immunocompromised patients may experience persistent MPXV infection (Contag et al., 2023; Rousseau et al., 2023), long-term use of a single antiviral agent could heighten the likelihood of resistance, highlighting the potential benefits of combination therapy for these individuals.

However, while combination therapies can overcome resistance to single agents, they may also increase the risk of toxicity compared with monotherapy. For these reasons, further studies and research are needed to develop new, specific antivirals for the treatment of Mpox.

## 7 Smallpox and Mpox vaccinia virus vaccines

Protection against Mpox disease is achieved by vaccination with vaccinia viruses that are protective also against smallpox disease. Vaccinia virus was initially used to vaccinate against smallpox disease at same time during the 19th century, when it replaced the cowpox virus for smallpox vaccination and became the preferred virus for it (Fenner et al., 1988; Parrino and Graham, 2006).

For about one century, until 1965 smallpox vaccination was unregulated and non-standardized and different strains of vaccinia virus and methods of storage and application were used. The vaccinia virus (VACV) strains New York City Board of Health (NYCBH), EM-63, and Lister/Elstree, were predominantly used during the early smallpox eradication campaign (Fenner et al., 1988).

In 1968, the WHO recommended the use of either the NYCBH strain or the Lister strain in the worldwide eradication campaign. From 1968 to 1971 the Lister strain became the most widely used vaccine worldwide (Rosenthal et al., 2001).

The vaccine used for eradication was grown in the skin of calves or other animals including sheep, buffaloes and rabbits (Committee for Proprietary Medicinal Products, 2002). After the eradication of smallpox, declared by the Health World Organization in 1980, the remaining licensed vaccines using the NYCBH strain were the lyophilized Dryavx (Wyeth), and liquid Wetvax (Aventis Pasteur). Dryvax was used in 2002 in two United States vaccination programs. Both programs reported similar rates of expected serious adverse events and in addition, abnormally high rates of cardiac related adverse events (Cassimatis et al., 2004; Poland et al., 2005).

The first-generation smallpox vaccine was reactogenic. Up to 40% of vaccinees experienced systemic symptoms including fever, myalgia, malaise and headaches. Moderate to severe complications that included generalized vaccinia, eczema vaccinatum, progressive vaccinia, post-vaccination encephalitis or encephalopathy and death were estimated to be 1–250 cases per million primary vaccinations, although the rates varied depending on the strain used, age groups (Jacobs et al., 2009). In general, among the vaccinia strains used for worldwide eradication, Dryvax^®^, was considered the safest, having the fewest adverse events (Kretzschmar et al., 2006).

In summary first generation smallpox vaccines were highly efficient as demonstrated during the Smallpox eradication campaign but they were relatively high reactogenic with a wide range of contraindications (Wiser et al., 2007).

The production of vaccine in live animals had important limitations due to contaminations by bacteria and adventitious agents, and to the presence of potentially allergenic animal proteins (Murphy and Osburn, 2007).

To overcome these limitations a second-generation vaccinia virus vaccine (VACV), named ACAM2000 (Novartis, formerly ACAMbis) was generated. It was a replication competent vaccine developed from a single clonal strain of Dryvax, that was propagated in Vero cell culture (Nalca and Zumbrun, 2010). ACAM2000 was approved by the FDA in 2007. Preclinical and clinical trials reported in 2008 demonstrated that ACAM2000™ had comparable immunogenicity to that of Dryvax^®^ and caused a similar frequency of adverse events.

To increase the safety of smallpox vaccine, third-generation attenuated VACVs were developed. Attenuated VACVs were obtained through sequential passages of the virus in tissue culture cells from alternative hosts (McCurdy et al., 2004). Modified Vaccinia Ankara (MVA) is an attenuated replication deficient poxvirus generated by more than 500 serial passages of vaccinia virus in chicken embryo fibroblast. Following the acquisition of multiple deletions and mutations, it lost the capacity to replicate in human cells (Drexler et al., 1998; Meyer et al., 1991). MVA-BN, that is derived from the MVA strain, is a further attenuated MVA strain. Due to the lack of replication competence in mammalian cells MVA-BN has a favorable safety profile for individuals including those with atopic dermatitis and immunodeficiency.

No signal for inflammatory cardiac disorders was identified throughout the MVA-BN development program. This is in sharp contrast to the older, replicating vaccinia smallpox vaccines, which have a known risk for myocarditis and/or pericarditis in up to 1 in 200 vaccinees (Volkmann et al., 2021).

## 8 Effectiveness and immunogenicity of smallpox vaccines against Mpox

Data from the active surveillance program for Mpox supported by the World Health Organization during 1980–1986 in the Democratic Republic of Congo provided the first evidence that smallpox vaccines protected against MPXV infection. This program, started after the official discontinuation of smallpox vaccination in 1980 and aimed to predict the future of Mpox dynamics in the absence of mass vaccination, showed a vaccine effectiveness of about 85% (Fine et al., 1988). The assessment of the risk of MPXV infection in the same endemic areas, performed about 25 years later demonstrated that people who were vaccinated with smallpox vaccine had a 5.2-fold lower risk of Mpox than the unvaccinated individuals confirming the protective role of smallpox vaccination. In addition, this and other studies reported that vaccine induced immunity was long lasting (Hammarlund et al., 2005; Rimoin et al., 2010).

Although mass vaccination with smallpox vaccine is not recommended during Mpox outbreak, during the multi-country Mpox outbreak in 2022, primary preventive vaccination was recommended for individuals at high-risk of exposure. They included gay, bisexual or MSM individuals with multiple casual sexual partners, sex workers; health workers and laboratory personnel working with orthopoxviruses. In addition, post-exposure preventive vaccination (PEPV) was recommended for contacts of cases, ideally within 4 days of first exposure (and up to 14 days in the absence of symptoms). Vaccination was used to interrupt human to human transmission, protect vulnerable individuals and minimize zoonotic transmission (World Health Organization, 2025a).

The vaccines considered in the response to the outbreak were, ACAM2000, MVA-BN, and LC16.

All of them, were developed against smallpox and while ACAM2000 was a second-generation vaccine, MVA-BN and LC16 were third-generation vaccines. MVA-BN was licensed as a vaccine against Mpox in humans in Canada (known as Imvamune) and the United States (known as JYNNEOS) and was approved by the European Medicines Agency under special circumstances (known as Imvanex), while LC16m8, a strain derived from the Lister strain of vaccinia virus, is approved for the prevention of Mpox in Japan. The efficacy of these vaccines could not be tested in randomized placebo- controlled clinical studies because of the infrequency of outbreaks. Evaluation of the protective effect of ACAM2000 and MVA-BN in a monkeypox model of infection in cynomolgus macaques showed that animals receiving either a prime and boost of Imvamune or a single immunization with ACAM2000 were protected completely from severe and/or lethal infection. Both antibody and cell-mediated immune responses were stimulated, and similar high titers of neutralizing antibodies were detected following the second dose of MVA-BN and one dose of ACAM2000 (Hatch et al., 2013) (Table 2).

**TABLE 2:** Summary of smallpox-derived vaccines evaluated for Mpox prevention.

Vaccine name	Generation	Strategy	Effectiveness	Use for MPXV	Limitations	References
Dryvax	First	Live replicating vaccine	Highly effective	No longer in use	Low safety profile	Kretzschmar et al. (2006), Cassimatis et al. (2004), Poland et al., 2005
ACAM2000	Second	Live replicating vaccine derived from Dryvax, grown in Vero cells	Similar effectiveness of Dryvax	Approved for MPXV in the USA	Low safety profile	Nalca and Zumbrun (2010), Jacob-Dolan et al. (2024)
MVA-BN (Imvamune, Imvanex, JYNNEOS)	Third	Live attenuated non-replicating vaccine	VE 33%–87% (1 or 2 doses), reduces hospitalization	Approved for MPXV in USA, Canada, and EU	Effectiveness partially known	Drexler et al. (1998), Meyer et al. (1991); Jacob-Dolan et al. (2024)
LC16m8	Third	Live attenuated vaccine	Preclinical efficacy in animal models	Approved for MPXV in Japan	Effectiveness largely unknown;	World Health Organization (2025b)
Ad35-subunit (L1R/B5R etc.)	Third (experimental)	Adenoviral vector subunit vaccine	Partial protection in animal models	Experimental	Effectiveness and safety unknown	Jacob-Dolan et al. (2024)

The table categorizes vaccines by generation, immunisation strategy, documented effectiveness, and limitations. It highlights historically used first-generation live replicating vaccines (e.g., Dryvax), second-generation cell culture–derived live vaccine (ACAM2000), and third-generation attenuated virus vaccines (MVA-BN/JYNNEOS, LC16m8). Effectiveness data and current MPXV usage are included alongside in-text references supporting these points.

More recently the comparison of the immunogenicity and protective efficacy of MVA-BN, ACAM2000 and Ad35 vector-based subunit (L1R/B5R or L1R/B5R/A27L/A33R) vaccine in a Rhesus macaque model of MPXV infection demonstrated that all vaccines provided robust protection against a high dose of intravenous MPXV challenge with the 2022–2023 outbreak strain (Jacob-Dolan et al., 2024). However, while a single dose of ACAM2000 provided a near complete protection, incomplete protection was achieved by the MVA-BN and the Ad35-subunit vaccines. During the clade IIb global spread, clinical studies have evaluated the MVA-BN vaccine effectiveness (VE) for Mpox, providing different estimates. The studies were performed in different countries and used various designs. The adjusted VE estimates against Mpox disease ≥14 days after pre-exposure vaccination with a single dose of MVA-BN were between 35% and 86% (Brousseau et al., 2024; Dalton et al., 2023; Deputy et al., 2023; Mason et al., 2024; Navarro et al., 2023; Payne et al., 2022; Ramchandani et al., 2023; Rosenberg et al., 2023; Wolff Sagy et al., 2023). For two doses of pre-exposure vaccination the VE estimates ranged from 66% to 90% (Dalton et al., 2023; Deputy et al., 2023; Payne et al., 2022). Crude VE estimates ranged from 33% to 84% and 57%–87% for one or two doses of MVA-BN respectively. Additionally, persons with Mpox who had received one or two doses of MVA-BN had a lower risk of hospitalization and decreased severity of Mpox clinical manifestation compared to unvaccinated individuals (Granskog et al., 2025; Guagliardo et al., 2024; Hazra et al., 2024; Schildhauer et al., 2023).

Data on LC16m8 vaccine effectiveness against Mpox are limited. A single immunization of LC16m8 induces robust cellular immune responses and broad neutralizing antibodies in immunocompetent and HIV infected individuals (Okumura et al., 2025). At present, based on vaccine effectiveness and safety profile MVA-BN represents the most promising candidate for broad use compared to ACAM2000 and LC16m8.

The Rhesus macaque model of monkeypox infection was also adopted to characterize the protective immune responses induced by vaccinia virus vaccine (Edghill-Smith et al., 2005). In this model, animals were vaccinated with the first-generation smallpox vaccine Dryvax by scarification and challenged 4 weeks later with monkeypox virus. The contribution of humoral or cell mediated immune response was assessed by antibody depletion of B cells or CD8 T cells in groups of animals. Results of this study showed that depletion of B cells, but not CD8^+^ T, affected the production of neutralizing antibodies and abrogated the vaccine induced protection from a lethal intravenous challenge with monkeypox virus demonstrating that the antibodies induced by smallpox vaccine are necessary and sufficient for protection against monkeypox virus (Edghill-Smith et al., 2005). Although there are not data on the same cohort, by combining the available immunogenicity data of MVA-BN to the corresponding vaccine effectiveness, a weak but significant association between antibody titers and vaccine effectiveness against Mpox could be demonstrated providing further support to the assumption that antibody levels may represent a correlate of protection (Berry et al., 2024). However, the recent assessment of MPXV neutralizing antibodies following two-shot MVA-BN immunization in non-primed individuals revealed that such antibodies were detected only in 63% of sera 4 weeks after the second MVA-BN vaccine dose and that in general, neutralizing antibodies showed a little increase after the second dose (Zaeck et al., 2023). At variance evaluation of the T cell memory response in convalescent and MVA-BN vaccinated individuals, using vaccinia virus infected cells or MPXV specific peptides, demonstrated that both individuals were able to mount a significant anti-MPXV T cell response with similar magnitude of CD4^+^ and CD8^+^ virus specific T cell responses, although T cells elicited by MPXV infection showed increased cytotoxicity and activation, a more expanded TCR repertoire and high potential to migrate to the site of infection compared to vaccine induced T cells suggesting that cellular immunity could be relevant (Chen et al., 2025).

## 9 Discussion

The large-scale outbreak of MPXV prompted the WHO to declare a public health emergency of international concern in July 2022, which is still in effect today, emphasising the need for continued surveillance of emerging pathogens (World Health Organization, 2024b), and underscores the importance of understanding the etiopathogenetic mechanisms and genomic evolution underlying the rapid spread of the MPXV.

Data sharing from MPXV strains isolated in various geographic regions has revealed an increased mutation rate, likely driven by APOBEC3-mediated mutagenesis, with a high concentration of single nucleotide polymorphisms (SNPs) found in the inverted terminal repeat (ITR) regions (Brinkmann et al., 2023; O’Toole et al., 2023). Although the phenotypic effects of recent MPXV mutations are not yet fully understood, the observed genomic changes likely underlie the virus’s increased transmissibility, atypical clinical features, and expanded geographic spread. Monitoring these evolutionary trends is essential for anticipating future outbreaks and informing public health responses.

The 2022 outbreak also highlighted the high rate of sexual transmission of Mpox: in non-endemic countries, an estimated 94% of MSM (Li et al., 2023). This epidemiological evidence supports the recommendation of targeted vaccination strategies for this population, as implemented in several countries (United States, Democratic Republic of the Congo and several neighbouring countries in Central and Eastern Africa, EU/EEA countries) (Centers for Disease Control and Prevention, 2025c; European Centre for Disease Prevention and Control, 2025; UNICEF, 2024).

Another critical aspect that emerged during the outbreaks starting in 2022 is the lack of specific effective antiviral therapies for Mpox. All drugs used in clinical settings have been FDA-approved for the treatment of smallpox, such as cidofovir and tecovirimat, or for other double-stranded DNA viruses. Unfortunately, clinical trials of tecovirimat conducted in the Democratic Republic of the Congo showed only limited efficacy in reducing Mpox-related lesions (PALM007 Writing Group et al., 2025; Van Dijck et al., 2024).

Cidofovir has been employed both systemically and topically, especially in severe cases, although concerns remain regarding its nephrotoxicity. Additionally, chemical compounds, such as the tecovirimat prodrug NIOCH-14, have demonstrated antiviral activity *in vitro*, and may offer potential future therapeutic options (Mazurkov et al., 2016).

Although some studies have identified peptides with potential antiviral activity against Mpox (Miah et al., 2022), these findings remain preliminary, and no clinical trials have been initiated to date. Recently, the viral resolvase enzyme has emerged as a promising therapeutic target. Mahoney and colleagues identified small molecules capable of inhibiting the resolvase of both vaccinia virus and MPXV *in vitro*, supporting its potential as an antiviral target against orthopoxviruses (Mahoney et al., 2025).

The ongoing epidemic should serve as an impetus for the development of MPXV-specific antiviral drugs with greater efficacy, as well as for the optimisation of more effective prophylactic strategies in terms of protective immunity.

Although tecovirimat is an approved and clinically effective antiviral, the range of available therapeutics targeting MPXV-encoded proteins remains limited, and the potential for the emergence of drug-resistant viral strains is a significant concern. These considerations underscore the urgent need to develop alternative and complementary therapeutic strategies. Among these, host-targeted antivirals (HTAs) have emerged as a promising and complementary therapeutic approach. Unlike direct-acting antivirals (DAAs), HTAs modulate host cell pathways involved in the viral life cycle or immune escape. This approach offers several advantages, including a lower risk of resistant mutants emerging due to the evolutionary stability of host targets; broad-spectrum antiviral potential; and additive or synergistic activity in combination with DAAs. Host-targeted strategies, successfully applied to other viruses, could also be exploited against MPXV; however, their clinical translation is still hindered by challenges such as cellular toxicity, the need for high selectivity to minimize off-target effects, pharmacokinetic optimization, and the lack of robust animal models that accurately reproduce human MPXV infection.

To date, vaccination remains the most effective form of prevention against Mpox. Among the available options, the non-replicating attenuated vaccine MVA-BN is currently the preferred one due to its safety profile and effectiveness.

Despite the availability of effective preventive vaccines, the absence of FDA-approved antivirals specifically targeting MPXV underscores the critical need for the development of dedicated therapeutic agents to improve clinical management and outbreak preparedness.

The Mpox outbreaks were caused by different virus strains, the first one by MPXV clade IIb, was characterized mainly by sexual transmission and it occurred in countries where the MPXV infection is not endemic. The second outbreak started in the Democratic Republic of Congo and rapidly spread to surrounding countries such as Uganda, Zimbabwe, the Central African Republic, Burundi, Rwanda, Kenya, underlining the importance of a rapid concerted response from various states to achieve concrete results. The management of the COVID-19 pandemic has taught us a lot in improving strategies and preparedness in health emergency situations: 1) an effective surveillance systems is based on a quickly identification of pathogens, 2) adequate information can improve adherence or preventive measures, 3) technical assistance and training for healthcare resource workers is indispensable in low-resource setting, 4) equitable distribution of vaccines support limit the diffusion of diseases and deaths (Adebiyi, 2025). Importantly, COVID-19 has also reshaped paradigms of vaccine deployment and pharmacological response, offering valuable insights for Mpox. In particular, integrating genomic surveillance with clinical and pharmacological data can reveal how viral evolution influences vaccine performance, drug response, and resistance development. Applying this approach to Mpox could support the optimization of therapeutic strategies, early detection of resistance, and the development of novel antivirals, ultimately improving both clinical management and public health interventions.

## 10 Conclusions

Recent Mpox outbreaks have highlighted how new variants can lead to different clinical manifestations, with varying severity and adaptation to humans, while also unveiling the limitations of currently used treatments. Although the genomic features of these lineages do not appear to affect treatment efficacy, monitoring the evolutionary dynamics within lineages remains essential for the development, evaluation, and efficacy of new antiviral compounds against Mpox. Furthermore, vaccination remains the most effective form of prevention against Mpox. Among the available options, the non-replicating attenuated vaccine MVA-BN is currently the preferred one due to its safety profile and effectiveness. However, even for MVA-BN, issues related to the high reactogenicity, the incomplete immunity, the uncertainty of cross protection and of the durability of vaccination exist. The solution of these issues drives the research aimed to improve or develop new Mpox vaccine strategies.
